# The Effect of Surface Nanometre-Scale Morphology on Protein Adsorption

**DOI:** 10.1371/journal.pone.0011862

**Published:** 2010-07-29

**Authors:** Pasquale Emanuele Scopelliti, Antonio Borgonovo, Marco Indrieri, Luca Giorgetti, Gero Bongiorno, Roberta Carbone, Alessandro Podestà, Paolo Milani

**Affiliations:** 1 Interdisciplinary Centre for Nanostructured Materials and Interfaces (CIMaINa) and Physics Department, Università degli studi di Milano, Milan, Italy; 2 Micro and Nano Fabrication Platform, Fondazione Filarete, Milan, Italy; 3 Department of Experimental Oncology, European Institute of Oncology Campus IFOM-IEO, Milan, Italy; 4 Tethis srl, Milan, Italy; Massachusetts Institute of Technology, United States of America

## Abstract

**Background:**

Protein adsorption is the first of a complex series of events that regulates many phenomena at the nano-bio interface, e.g. cell adhesion and differentiation, *in vivo* inflammatory responses and protein crystallization. A quantitative understanding of how nanoscale morphology influences protein adsorption is strategic for providing insight into all of these processes, however this understanding has been lacking until now.

**Methodology/Principal Findings:**

Here we introduce novel methods for quantitative high-throughput characterization of protein-surface interaction and we apply them in an integrated experimental strategy, to study the adsorption of a panel of proteins on nanostructured surfaces. We show that the increase of nanoscale roughness (from 15 nm to 30 nm) induces a decrease of protein binding affinity (≤90%) and a relevant increase in adsorbed proteins (≤500%) beyond the corresponding increase of specific area. We demonstrate that these effects are caused by protein nucleation on the surface, which is promoted by surface nanoscale pores.

**Conclusions/Significance:**

These results show that the adsorption of proteins depends significantly on surface nanostructure and that the relevant morphological parameter regulating the protein adsorption process is the nanometric pore shape. These new findings improve our understanding of the role of nanostructures as a biomaterial design parameter and they have important implications for the general understanding of cell behavior on nanostructured surfaces.

## Introduction

Surface physical properties have a relevant role in regulating the interaction between biomaterials and biological systems [1]. In particular surface nanoscale morphology profoundly influences cell adhesion, spread, growth and differentiation [1]–[7]. This concept has sparked new research approaches, where the control of surface nanostructure is used as a biomaterial design parameter to regulate cell functions, such as stem cell differentiation for tissue engineering *in vitro* and *in vivo* [8]–[11]. Biomaterial surfaces in biological environments are rapidly coated by proteins that mediate the interaction between the biomaterial and cells [12]–[14], regulating the final cell behaviour through complex signalling pathways [15]. Therefore, the quantitative characterization of how nanoscale surface features determine the amount, structure and distribution of adsorbed proteins is necessary for understanding cell-nanostructured surface interaction [8], [9], [16]–[19]. This knowledge of the protein adsorption process on nanostructured surfaces is also relevant to many research fields such as tissue regeneration [8]–[10], drug delivery [20]–[23], prosthetics [16], nanotoxicology [24], heterogeneous nucleation [25], [26], biosensing [27], [28] and therapeutic micro- and nano-devices [29], [30].

Several attempts have been made to characterize the influence of nanoscale morphology on protein adsorption. Studies on nanoparticles in solution have provided insights into protein-nanoparticle interactions [14], [31], [32], highlighting the role of nanoparticle curvature in the folding of adsorbed proteins [33]. These results, however, cannot be directly transferred to nanostructured biomaterial surfaces, being radically different systems. In addition, previous experiments specifically designed to characterize protein adsorption on nanostructured surfaces resulted in quite inconsistent observations [34]–[37]; some reports showed no influence of the morphology at the nanoscale level [34], [35], while others presented an increase of the amount of adsorbed proteins when nanoscale surface roughness increased [36], [37]. This incoherent picture arises from the fact that protein adsorption on nanostructured surfaces has never been fully quantitatively characterized, both because of the remarkably large number of parameters affecting the adsorption process, and because of the lack of suitable tools for studying adsorption on rough surfaces. A full characterization of protein adsorption on a nanostructured surface should consist of a controlled variation of the following parameters: nanoscale morphology, protein concentration and protein type. Varying surface morphology requires the production of nanostructured surfaces with exactly the same chemical composition, and a measurable change in morphology (usually quantified as a change of surface roughness) in order to isolate its role in the adsorption process. Changing protein concentrations allows producing adsorption isotherms that, in turn, are used to calculate protein binding affinity. Proteins have remarkably diverse characteristics (e.g. in terms of charge, size, solubility); it is therefore crucial to characterize the adsorption process with several proteins in order to draw definitive conclusions. In this framework, traditional quantitative techniques used to measure the amount of adsorbed proteins on surfaces, such as quartz crystal microbalance (QCM) and ellipsometry, fail in giving reliable results on rough surfaces [36], [38], making the analysis of the multi-parameter phase space that characterizes the adsorption process even more complex.

In order to overcome these difficulties, and to correlate adsorption data with morphological surface parameters, we implemented an innovative integrated experimental strategy. First, we used supersonic cluster beam deposition (SCBD) to produce samples of nanostructured titania (ns-TiO_x_) with gradually increasing surface roughness. Second, we developed and applied novel quantitative high-throughput methods, based on microarray technology and confocal microscopy, to ns-TiO_x_. Using this fully parallel approach, we studied the amount of adsorbed protein as a function of protein concentration on several nanostructured surfaces and for a panel of proteins. Then, driven by our results, we used atomic force microscope (AFM) for characterizing the structure of the adsorbed layer at the nanometre scale.

## Results and Discussion

### Surface synthesis

**Figure 1:**
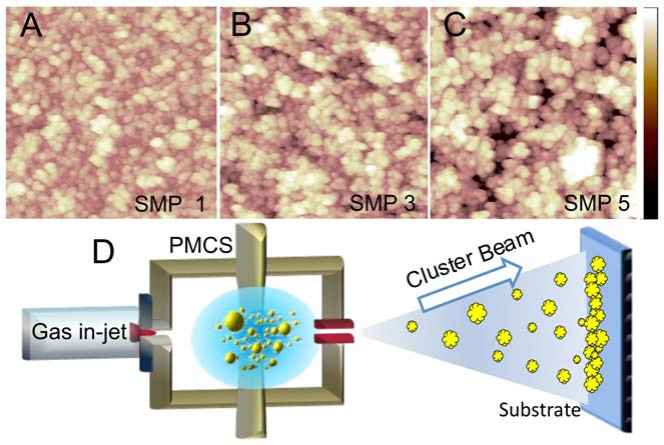
Nanostructured surface synthesis. (A–C) AFM images of surface morphology for sample 1 (SMP1, A), sample 3 (SMP3, B) and sample 5 (SMP5, C). Colour scale range is 0–120 nm (black to white). (D) Schematic view of the supersonic cluster beam deposition (SCBD) apparatus equipped with a pulsed microplasma cluster source (PMCS).

Using a SCBD apparatus, equipped with a pulsed microplasma cluster source (PMCS), we produced five different groups of ns-TiO_x_ samples with increasing film thickness, returning five different morphologies characterized by a root-mean-square (RMS) surface roughness, ranging from 15 nm to 30 nm (Fig. 1A, 1B, 1C and Table 1). These films are ideal tools for investigating the role of nanoscale roughness in protein adsorption. Ns-TiO_x_ films are made by the deposition of nanometric clusters onto glass slides (Fig. 1D), and they are characterized by a random nanoscale roughness mimicking those of many biological systems [8], [39]. Since cluster deposition is performed in ballistic regime, the film roughness is varied from 15 to 30 nm by changing the thickness of the deposited films, without changing the surface chemistry [39] (Fig. S1). Additionally, ns-TiO_x_ films showed a high biocompatibility with primary and cancer cell lines [40], [41], a good efficacy in protein immobilization [42], and the possibility of tailoring their physical and chemical properties by controlling the cluster assembling parameters [39], [43]. After annealing at 250°C films are hydrophilic (contact angle cosθ = 0.9) [39]. Ns-TiO_x_ films interact with proteins via non-specific interaction (electrostatic, hydrophobic and Van der Waals interactions) and via specific interaction (covalent bond between protein acidic side chains and undercoordinated titanium atoms on the surface) [42].

**Table 1:** Nanostructured surface morphology.

Sample ID	T [nm]	R [nm]	SA
SMP 1	50	15.1±0.2	1.54±0.09
SMP 2	100	19.2±0.2	1.62±0.04
SMP 3	150	22.1±0.2	1.79±0.11
SMP 4	200	25.1±0.1	1.87±0.02
SMP 5	340	29.5±0.8	1.95±0.03

Roughness and specific area of the five groups of produced samples measured with the Atomic Force Microscope. T is film thickness, R is RMS roughness and SA is specific area.

We used AFM for characterizing surface morphology and measuring surface roughness and specific area (Table 1). We also performed a numerical simulation comparing the result of the convolution of AFM tip and protein-like probes with ns-TiOx surfaces, showing that AFM reliably measures the effective specific area available for protein adsorption (Fig. S2, S3 and Supplementary Discussion S1).

### Protein adsorption isotherms

**Figure 2:**
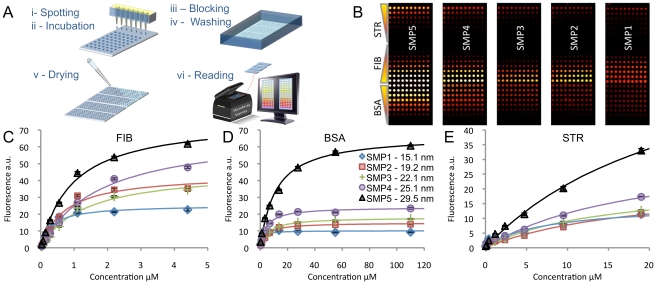
PSIM applied to nanostructured surfaces. (A) Sketch of the 6 steps PSIM protocol. We verified the feasibility of this approach with specific assays. We demonstrated that the fluorescent signal is proportional to the amount of adsorbed proteins and that the fluorescent marker used in our experiment does not influence the protein-surface interaction process (Fig. S4). We tested PSIM protocol repeating the same experiment 3 times on a group of twin samples obtaining highly reproducible results (Fig. S5). Finally, using Fluorescence Recovery After Photobleaching (FRAP), we also verified that the adsorbed proteins remain immobilized on the surface after adsorption (Fig. S6). (B) Raw data obtained by reading the slides of the PSIM experiment in which 8 concentrations of BSA, fibrinogen and streptavidin were spotted in 10 replicates on the 5 nanostructured titania samples, with different surface morphology. (C) Adsorbed Fibrinogen, (D) BSA and (e) Streptavidin as function of protein concentration (adsorption isotherms) for 5 ns-TiOx samples (samples 1–5 in Table 1) with different surface roughness. PSIM allowed obtaining adsorption isotherms on nanostructured surfaces for the first time. Data are fitted with Langmuir isotherm in order to calculate saturation uptake and binding affinity. Error bars correspond to standard deviation of the 10 replicates.

In order to study the protein adsorption process on rough films, we developed a new high-throughput approach called protein-surface interaction microarrays (PSIM, Supplementary Fig. S4, S5, S6 and Discussion S1), which is designed to yield protein adsorption isotherms for a panel of proteins on several surfaces simultaneously. The PSIM protocol consists in spotting small-volume droplets (30 nl) of fluorescent labeled proteins, diluted in a wide range of concentrations, on the sample surfaces under investigation. After incubation, blocking, washing and drying, the amount of adsorbed proteins is evaluated by reading the fluorescent signal with a commercial microarray scanner (Fig. 2A). For investigating the role of nanoscale morphology in protein adsorption we performed a PSIM experiment spotting 8 different concentrations of bovine serum albumin (BSA), fibrinogen and streptavidin (10 replicates per concentration) on the 5 titania nanostructured surfaces described above (Fig. 2B, Supplementary Discussion S1 for proteins characteristics). In this experiment we studied 1,200 protein-surface interactions obtaining protein adsorption isotherms on nanostructured surfaces (Fig. 2C, 2D, 2E). The Langmuir isotherm model, the most widely used protein adsorption model [44], adequately reproduces our experimental data for all the tested proteins (Fig. 2C, 2D, 2E):
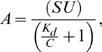
where A is the amount of adsorbed proteins; C is the protein concentration; SU represents, in fluorescence units, the surface saturation uptake, which is the maximum amount of protein that the surface can load; and K_d_ is the concentration, corresponding to half of the maximum of the adsorption curves, and it is inversely proportional to the protein binding affinity [33], [44].

**Figure 3:**
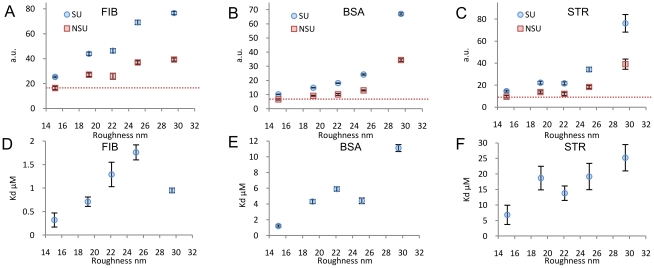
Adsorption data as function of surface roughness. (A–C) Adsorption saturation uptake (SU) and normalized saturation uptake (NSU) as function of surface roughness for fibrinogen (A), BSA (B) and streptavidin (C). The dotted red line shows the expected NSU trend if saturation uptake were proportional to specific area. Protein adsorption is significantly increased beyond the corresponding increase of specific area when surface roughness increases. (D–F) K_d_ as a function of surface roughness for fibrinogen (D), BSA (E) and streptavidin (F). Even if samples have identical surface chemistry, the increase of surface roughness causes an apparent increase of K_d_, which corresponds to a decrease in protein binding affinity. Error bars correspond to parameters standard deviation.

PSIM results show that surface nanoscale morphology drastically influences the amount of adsorbed proteins. The saturation uptake significantly increases as nanoscale roughness increases. Surprisingly, when changing surface roughness by 15 nm, the saturation uptake increases up to 600%, depending on the protein used (Fig. 3A, 3B, 3C). Results also demonstrate that the adsorption mechanism follows different modalities than those expected, since the effect produced by increasing roughness is not justified by mere geometry, i.e. the creation of new adsorption sites. If this were the case, the amount of adsorbed proteins should increase linearly at most, as a function of the sample specific area, because of the consequent increase of adsorption sites. Moreover, since samples have identical surface chemistry, binding affinity would be expected to remain constant when nanoscale morphology changes. However, measured SU is not directly proportional to the number of adsorption sites on the surface; in fact, the normalized saturation uptake (NSU), defined as the SU divided by the sample specific area, follows an evident growing trend for all the considered proteins (Fig. 3A, 3B, 3C). This shows that the increase in protein adsorption is more than linear as a function of the increase of disposable adsorption space on the surface. Additionally, changing surface morphology causes an increase in K_d_, representing a reduction of the protein binding affinity of up to 90%. K_d_ increases almost linearly as a function of nanoscale roughness, with few exceptions (Fig. 3D, 3E, 3F). A simple increase in the number of adsorption sites is not enough to explain these data.

### Adsorbed Proteins Quantification

**Figure 4:**
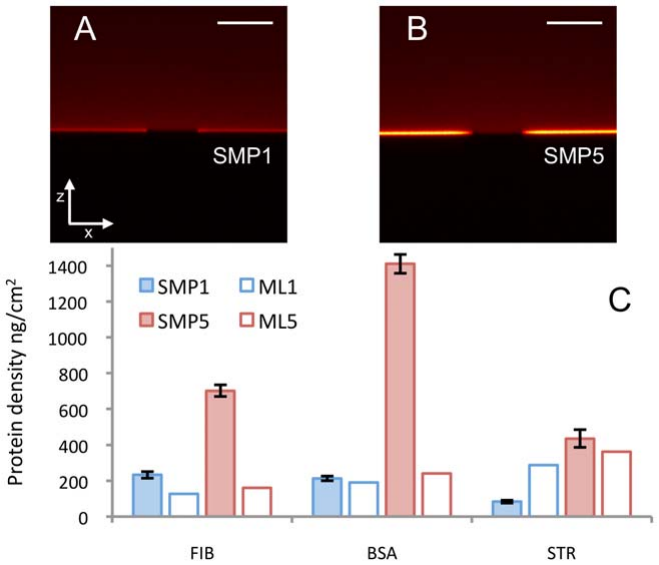
Quantification of the amount of adsorbed proteins. FPQ images for the adsorption of BSA on sample 1 (A) and sample 5 (B) at 27.5 µM concentration. The central part of the adsorbed layer was bleached for measuring the background signal that needs to be subtracted from the layer signal (see Fig. S7 and Supplementary Discussion S1). FPQ confirms PSIM results: at 27.5 µM concentration the same ratio between the amount of adsorbed protein on sample 1 and sample 5 was measured (5.1±0.4 measured with FPQ and 4.9±0.2 measured with PSIM). This result was used to calibrate the PSIM experiment (see Materials and Methods). Bar is 15 µm. (C) Results of the quantification of the saturation uptake on samples 1 and 5 for fibrinogen (FIB), BSA and streptavidin (STR). Quantitative results are compared to the theoretical amount of adsorbed proteins expected for a protein monolayer calculated using the RSA model and considering the specific area of the sample 1 (ML1) and sample 5 (ML5). Error bars correspond to standard deviation of 3 experiment replicates.

To further investigate these effects, we validated and quantified the former PSIM experiment with a complementary new approach, fluorescence photobleaching quantification (FPQ). FPQ consists in imaging the adsorbed protein layer in the plane perpendicular to the surface (xz plane) with a confocal microscope, immediately after photobleaching part of the layer. The bleached zone allows accurate measurement of the intensity of the background fluorescence, caused by fluorescent proteins in solution. The background has a complex shape because it is affected by optical aberrations [45] (Fig. S7). The signal of the adsorbed layer is isolated by subtracting the background signal from the raw signal. Additionally, because the concentration of proteins in the solution is known, the background is used to quantify the layer signal (Fig. S7, Supplementary Discussion S1). It is worth stressing that FPQ is a powerful tool *per se*, which, in principle, may be applied to any surface, and specifically to any rough surface. Fig. 4A and 4B show typical FPQ images for BSA adsorbed on samples 1 and 5 (the least and most corrugated surfaces in the previous PSIM experiment, respectively). FPQ detected the same non-linear adsorption enhancement effect that we observed with PSIM, with the same ratio between the amount of adsorbed proteins on samples 1 and 5 at high concentration (Fig. 4A, 4B). Quantitative analysis allows PSIM experiment calibration and the measurement of surface protein density (Fig. 4C). The comparison of quantitative results with theoretical values of monolayer coverage, based on the random sequential adsorption model [46], shows quite a high protein density on the surface. For BSA and fibrinogen, on the roughest samples, protein density is well beyond the theoretical monolayer (Fig. 4C). These results prove that the increase of surface roughness promotes protein-protein interaction on the surface, which may also induce the formation of multiple protein layers.

By exploiting the unique properties of PSIM, FPQ and ns-TiOx surfaces, we quantitatively characterized the adsorption process as a function of the main parameters of the system: surface morphology, protein concentration and protein type. By using a number of different proteins, we have obtained different layer densities, concentration ranges and SU and K_d_ trends, as a function of surface roughness. However, this full characterization highlights important experimental evidence that is common among the proteins tested: i) the amount of adsorbed proteins is increased significantly more than expected from the corresponding increase of specific area when surface roughness increases; ii) the increase of surface roughness causes a decrease of protein binding affinity; iii) on the rougher samples, adsorption results in high surface protein density and in the formation of protein multilayers (for BSA and fibrinogen).

### AFM analysis

**Figure 5:**
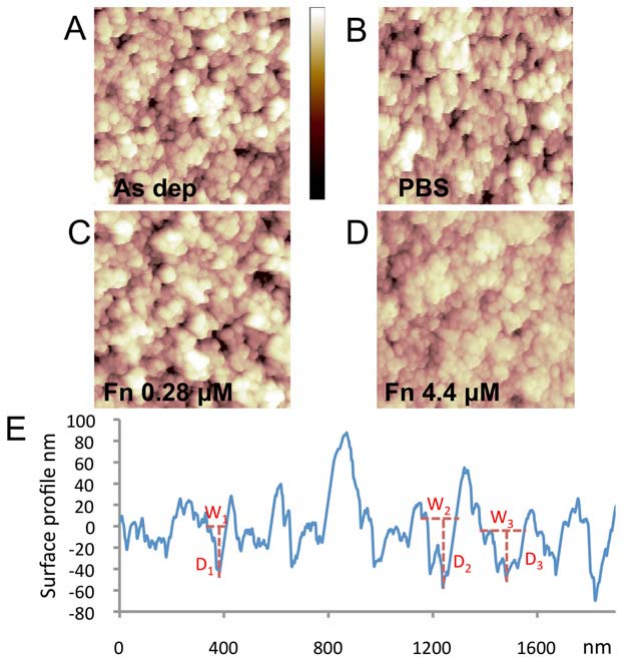
AFM images of surface morphology before and after fibrinogen adsorption. (A) As-deposited ns-TiOx sample with RMS roughness 26.2±0.1 nm. (B) Sample after PBS incubation, with RMS roughness 26.3±0.1 nm. (C) Sample after incubation with fibrinogen solution at 0.28 µM, a surface roughness of 25.8±0.1 nm shows that after adsorption at low concentration, surface morphology is not substantially changed. (D) Sample after incubation with fibrinogen 4.4 µM, which causes a remarkable surface flattening, resulting in a surface roughness of 21.2±0.1 nm. Colour scale range is 0–120 nm (black to white). (e) Surface profile of a typical ns-TiOx surface shows that the surface is characterized by nanometric pores with variable pore width and depth. The red dotted line indicates width (W) and depth (D) of some of the surface nanometric pores.

On the basis of these results, we performed AFM experiments in order to understand the mechanism through which surface morphology influences protein adsorption. We focused on fibrinogen and BSA, for which we detected the formation of multiple protein layers, and we produced ns-TiO_x_ samples with a RMS roughness of 26.2 nm. We measured the surface morphology of the as-deposited sample (i.e. *without* protein incubation) (Fig. 5A), of the same sample after incubation with phosphate-saline buffer (PBS) (Fig. 5B), and with fibrinogen at 0.28 µM (Fig. 5C) and 4.4 µM (Fig. 5D, the same experiment for BSA is presented in Fig. S8). After PBS incubation, as expected, the surface morphology remained unchanged. Also incubation with fibrinogen at 0.28 µM did not significantly affect the AFM surface morphology (Fig. 5C), in line with previously published results [34]. However, after fibrinogen adsorption at 4.4 µM, the surface morphology of the sample was markedly flattened, the sample having significantly lower surface roughness (21.2 nm compared to the original 26.2 nm, Fig. 5D).

**Figure 6:**
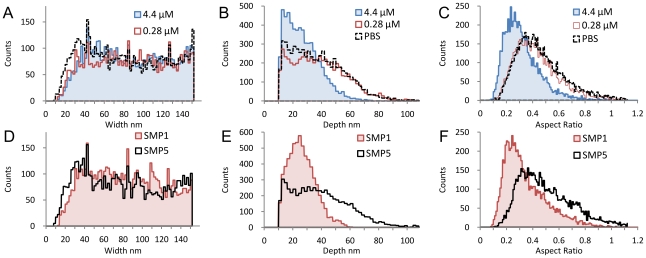
AFM quantitative image analysis of surface morphology. Analysis performed on samples synthetized for AFM experiment (RMS roughness 26.2±0.1 nm): (A) widths spectrum of pores after sample incubation with PBS, fibrinogen at 0.28 µM and 4.4 µM, the distribution is very similar before and after fibrinogen adsorption; (B) depth spectrum of pores after sample incubation with PBS, fibrinogen at 0.28 µM and 4.4 µM, depth distribution after adsorption at 4.4 µM is very different from the other two in the whole depth range; in the region between 40 nm and 100 nm, the population is completely depleted; (C) aspect ratio spectrum of pores after sample incubation with PBS, fibrinogen solution at 0.28 µM and 4.4 µM; for aspect ratios higher than 0.5, 75% of pores are filled, showing that nucleation preferentially occurs in pores with higher aspect ratio. Analysis performed on samples 1 and 5 used in previous FPQ and PSIM experiments: (D) width spectrum of pores for samples 1 and 5 as-deposited shows that increasing surface roughness does not substantially change the width distribution; (E) depth spectrum of pores for samples 1 and 5 as-deposited shows that the increase in surface roughness is related to the increase of the pores depth; (F) aspect ratio spectrum of pores for samples 1 and 5 as-deposited; the higher roughness of sample SMP5 results in an aspect ratio distribution with a significantly higher population for aspect ratio higher than 0.5.

Any surface section of a typical ns-TiO_x_ sample is characterized by nanometric pores of diverse depths and widths (Fig. 5E). We developed a quantitative method of AFM image analysis for statistically characterizing the depth and the width of each pore on the surface profile in Fig. 5 (see Methods S1 and Fig. S9, S10). Results are presented in Fig. 6A and 6B. The distributions of pore width (Fig. 6A) are very similar before and after adsorption, while the depth distribution after adsorption at 4.4 µM is very different from the others in the whole depth range (Fig. 6B). In fact, after adsorption at 4.4 µM, the depth of deep pores is remarkably reduced. In addition, the depth spectrum is substantially compressed to the lowest depth region, showing that part of the surface pores are filled or partially filled by proteins. These results demonstrate that the formation of multiple protein layers is localized in specific nanometric structures; in fact, part of the surface pores is filled by proteins that aggregate inside the pores. Importantly, we did not observe the same effect after incubation with fibrinogen at 0.28 µM (Fig. 6B) showing that aggregation inside the pore is concentration dependent. Further information can be extracted taking into account the aspect ratio distribution, defined as depth/width ratio (Fig. 6C). This shows that aggregation tends to occur preferentially in pores with aspect ratio higher than 0.5 (Fig. 6C), given that 75% of those pores were partially filled. This indicates that aggregation happens more frequently inside pores with higher aspect ratio. We have also obtained similar results with BSA, in which 75% of pores having aspect ratios greater than 0.4 are filled (Fig. S11).

The comparison of pore width, depth and aspect ratio spectra of the as-deposited samples 1 and 5 (Fig. 6D, 6E, 6F respectively) shows that the increase of surface roughness is correlated with the increase of pore aspect ratio. The two samples have indeed very similar pore width distributions (Fig. 6D), but sample 5 has a broader depth distribution with a significantly greater population in the higher depth region (Fig. 6E), which results in a wider aspect ratio distribution with an increased number of pores having high aspect ratio values (Fig. 6F). We calculated the total volume of pores having aspect ratios greater than 0.5, representing the available volume for the formation of protein clusters (Fig. S12). As nanoscale roughness increases, this volume increases beyond the corresponding increase of specific area, accounting for the similar trend followed by protein SU as a function of surface roughness (Fig. 3A, 3B and 3C). We also estimated the number of aggregation sites per µm^2^ and the mean number of proteins that form a protein cluster (Fig. S12). The change in surface roughness coincides with the increase in the number of protein clusterization sites, induced by the increase of the number of pores with aspect ratio higher than the threshold value.

### Conclusions

These results concur both with experimental data, showing protein crystallization on nano-porous materials [26], [27], and with performed simulation of phase change inside a nanometric pore [47]. They suggest that nanostructured surfaces promote the formation of protein aggregates because nanometric pores generate the conditions for protein nucleation inside the pores. Since the pore width is approximately the size of a few proteins and pores have high aspect ratios, a protein entering the pore may remain trapped, spending a longer time inside the pore when compared to the diffusion time [48]. During this dwelling time, other proteins can, in turn, be trapped inside the pore, resulting in a crowding effect that significantly reduces the mean protein-protein distance. When proteins are trapped inside the pore, the presence of adsorbed proteins on the pore walls may further contribute to the reduction of the mean protein-protein distance. These effects concomitantly participate to the formation of local supersaturation spikes and thus to protein nucleation inside the pores. Once the conditions for supersaturation do not hold anymore, i.e. when the pore is filled or partially filled, nucleation stops. This mechanism explains all the results obtained with PSIM and FPQ. By growing surface roughness, the number of nucleation sites increases. Furthermore, the volume available for nucleation grows beyond the increase of specific area (Fig. S12), causing a significant increase of the amount of adsorbed proteins (Fig. 3A, 3B and 3C), which may be even higher than expected for a full monolayer (Fig. 4C). The nucleation process also accounts for the observed increase of K_d_ (Fig. 3D, 3E and 3F). When increasing the pore depth (surface roughness), a higher concentration is needed to generate supersaturation inside the pores. Thus, the increase in K_d_ does not reflect changes in the chemical affinity of surface-protein interaction; rather, it reflects the formation of supersaturation spikes, which depends on solution concentration and pore shape distribution.

We have quantitatively characterized the role of nanoscale morphology in influencing protein adsorption, highlighting the mechanism that determines how proteins organize on nanostructured surfaces. Nanoscale morphology significantly increases the amount of adsorbed proteins, causing the formation of protein clusters in correspondence with surface nanometric pores. Proteins nucleate inside pores with aspect ratios higher than specific threshold values, which depend on the characteristics of each protein; we measured this threshold to be approximately 0.5 for fibrinogen and 0.4 for BSA in our system. These results define the role of nanoscale morphology as a biomaterial design parameter to control the amount of adsorbed proteins and the structure of the adsorbed layer, showing that the morphological parameter regulating the nucleation process is the nanometric pore shape distribution. This finding is highly significant for many applications where nanostructures interact with biological systems, for the understanding of cell-nanostructured surface interaction and for the general understanding of the nano-bio interface. The systematic quantification of protein-surface interaction has been made possible by the development of new, high-throughput and quantitative methods, allowing the analysis of protein adsorption onto nanostructured surfaces, and the comparison of up to 1,200 interactions in a single experiment. Moreover, these novel methods can be applied to any kind of surface, including polymers. They can therefore facilitate the screening of biomaterial libraries against panels of proteins, in the framework of combinatorial approaches, to optimize biomaterial performance [49]–[52].

## Materials and Methods

### Nanostructured Surface Synthesis by PMCS

Nanostructured TiO_x_ films were deposited by a supersonic cluster beam deposition (SCBD) apparatus equipped with a pulsed microplasma cluster source (PMCS). The PMCS operation principle is based on the ablation of a titanium rod by a helium plasma jet, ignited by a pulsed electric discharge. After the ablation, TiO_x_ ions thermalize with helium and condense to form clusters. The mixture of clusters and inert gas is then extracted in vacuum through a nozzle to form a seeded supersonic beam, which is collected on a set of 4 standard glass microscope slides (25 mm×75 mm) and 4 glass coverslips (diameter 15 mm) located in the beam trajectory. The clusters kinetic energy is low enough to avoid fragmentation and hence a nanostructured film is grown. Five different depositions were performed in order to produce five groups of samples with different morphologies, depositing different film thicknesses: 50 nm (sample 1, SMP1), 100 nm (sample 2, SMP2), 150 nm (sample 3, SMP3), 200 nm (sample 4, SMP4) and 340 nm (sample 5, SMP5). Samples were thermally annealed in air using a muffle furnace at 250°C, reached through a slow ramp and maintained for 24 hours.

### Sample morphology characterization with AFM

The investigation of morphology of the substrates was carried out in air using a Multimode AFM equipped with a Nanoscope IV controller (Veeco Instruments). The AFM was operated in Tapping Mode use single crystal silicon tips with nominal radius of curvature 5–10 nm and cantilever resonance frequency in the range of 200–300 kHz. Scan areas were 2µm×1µm with scan rates of 1.5–2 Hz. Sampling resolution was 2048×512. More details in Supplementary Methods S1.

### Protein Surface Interaction Microarrays

Alexa 647 labeled fibrinogen, BSA and streptavidin (Molecular Probes) were dissolved in PBS buffer (pH 7.4). at 4.4 µM, 110 µM and 19 µM concentration respectively. Protein concentration was measured using spectrophotometer. 8 sequential dilutions (1∶2) of the mother solution were prepared in a 96-well plate for the three proteins. 30 nL solution droplets were spotted on ns-TiOx slides using an automated spotter (BioDot AD3400) in an array format (24 lines with 10 replicates per line) at 75% controlled humidity in order to avoid drop evaporation. After spotting slides were incubated for 1h at room temperature and 75% humidity. After incubation slides were blocked 1 time in BSA 2% for 1 minute and washed 3 times in PBS for 1 minute and 3 times in bidistilled H_2_O for 1 minute. Slides were dried under gentle nitrogen flux. Fluorescence was then quantified using a microarray scanner PerkinElmer and images were analyzed using Scanarray Express software.

### Fluorescence Photobleaching Quantification

Ns-TiO_x_ coverslip samples were incubated with 400 µL of Alexa 647 BSA solution at 27.5 µM for 1h in a custom-made plastic incubation chamber. The adsorbed layer was then imaged in the xz plane using a Leica SP1 confocal microscope using He/Ne (633 nm) laser, 10% laser power, 63× oil immersion objective, 256×256 image resolution and 3× magnification. Part of the adsorbed layer was bleached in the xy plane using 100% laser power and 16×magnification factor. 3 images of the layer with the bleached zone were acquired immediately after the bleaching in the xz plane with previous settings. Images were analyzed using an ImageJ automated routine for layer signal and background estimation. The density of adsorbed proteins was calculated as follows: 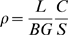, where ρ is the protein layer density, L is the intensity of the layer signal, BG is the intensity of the background signal in correspondence of the layer, C is protein concentration and S is the resolution area of the microscope in xy plane. PSIM experiment was quantified using the following relation for converting fluorescence units in absolute units: 
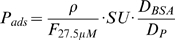
, where 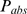 is the protein layer density in absolute units [ng/cm^2^], 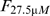
is the fluorescence intensity measured in the PSIM experiment at 27.5 µM, SU is the saturation uptake, D_BSA_ is the BSA degree of labeling and D_P_ is fibrinogen or streptavidin degree of labeling. The degree of labeling was measured by separately determining the protein and fluorophore molar concentrations of the conjugate, using absorbance measurements, and then expressing these concentrations as a ratio.

## Supporting Information

Supporting Discussion S1 The AFM measurement of the available area for protein adsorption, PSIM fluorescence linearity, protein immobilization, fluorescence photobleaching quantification and the properties of the proteins used in this study are discussed in further details.
(0.28 MB PDF)

Supporting Methods S1 Supporting Methods.
(0.13 MB PDF)

Figure S1 Ns-TiOx film growth. Schematic view of the film growth process as a function of the deposition time (film thickness). Changing film thickness is possible to regulate surface morphology without changing surface chemistry. This method allows varying surface roughness from 15 nm to 30 nm (Fig. 1 and table 1).
(0.53 MB PDF)

Figure S2 Simulation of the self-affine fractal ns-TiOx profile. Typical surface profile of: a) ns-TiOx sample 5 (experimental, 2 µm scale); b) SIM5_TIP5 simulated profile (SIM5) after convolution with AFM tip of radius 5 nm (2 µm scale); c) SIM5 simulated profile (2 µm scale); d) ns-TiOx sample 5 (experimental, 500 nm scale); e) SIM5_TIP5 simulated profile (SIM5) after convolution with AFM tip of radius 5 nm (500 nm scale); f) SIM5 simulated profile (500 nm scale). g) Experimental morphological parameters (left) compared with morphological parameters of simulated surfaces (center) and simulated surfaces after 5 nm tip convolution (right). The convolution of a AFM tip of radius 5 nm with simulated profiles returns a two dimensional surface area, S_A2D_, very similar to the experimental one, demonstrating that simulation faithfully reproduces experimental surfaces.
(0.17 MB PDF)

Figure S3 Surface-probes convolution results. a) The 2D surface area of simulated profiles of increasing roughness after convolution with AFM-like and protein-like probes of different radii (TIP 5 nm and 7 nm; PROT 3 nm, 5 nm, 7 nm and 10 nm). SA2D stands for the surface area of the simulated sample without any convolution. b) The relative difference between the specific area measured with AFM-like tips of radius 5 and 7 nm (left and right halves of the table) and the specific area measured with protein-like probes of different radii (mimicking the specific area available for adsorption to proteins of different dimensions). The comparison with the specific area of the not-convoluted profiles is also shown in the first raw. c) The available surface area for protein adsorption as a function of surface roughness for different probe radii. d) The relative difference between the ratios of surface areas of samples SIM5 and SIM1 measured with AFM-like and protein-like probes of different radii.
(0.03 MB PDF)

Figure S4 Fluorescence signal linearity. a) The sketch of the assay used to test the linearity of the fluorescent signal as a function of the amount of adsorbed proteins. The objective of the assay is to measure the fluorescent signal as a function of the part of labeled BSA in solution. b) Protein-surface interaction array composed of 4 sub-arrays; in each sub-array the protein concentration was kept constant (0.75 µM, 1.5 µM, 3 µM and 6 µM), while the part of labeled BSA in solution was varied from 0% to 100%. In each line the same BSA concentration is spotted in 10 replicates. c) Results of the PSIM validation experiment. Data follows a good linear trend for all the used concentrations, showing that the fluorescence signal is proportional to the amount of adsorbed proteins.
(0.16 MB PDF)

Figure S5 PSIM reproducibility. In order to test PSIM reproducibility we performed three independent experiments spotting 18 different fluorescently labelled BSA dilutions, in 10 replicates, on three different ns-TiOx samples (same thickness, 50 nm, resulting in a surface roughness of 15.0±0.1 and in a specific area of 1.56±0.1). The result of each experiment is compared with the mean of the three experiments (last bar of each concentration point). Error bar is the standard deviation of the 10 replicates for each experiment point. For the mean the error bar is the standard deviation of three experiments.
(0.03 MB PDF)

Figure S6 Fluorescence Recovery After Photobleaching for studying protein stability. Confocal microscope images of the adsorbed layer for sample 1 (a) just after the photobleaching of part of the adsorbed layer and and (b) 60 minutes after the photobleaching. (c) Fluorecence recovery after photobleaching as a function of time for sample 1. Confocal microscope images of the adsorbed layer for sample 5 (d) just after the photobleaching of part of the adsorbed layer and and (e) 60 minutes after the photobleaching. (f) Fluorecence recovery after photobleaching as a function of time for sample 5.
(0.18 MB PDF)

Figure S7 Fluorescence Photobleaching Quantification. a) A glass coverslip was passivated with BSA (without fluorescent label) in order to avoid the following adsorption of labeled proteins. Coverslip was then incubated with a solution of Fluorescent BSA 1 µM concentration. b) The the signal profile. The surface is passivated and the signal corresponds only to the BG signal, which has a complex shape because of PSF convolution and optical abberrations. The dashed line represents a step function, the expected BG shape without PSF convolution and optical aberrations. c) Image of the adsorbed layer on ns-TiOx sample incubated with fluorescent BSA 5 µM concentration. d) Image of the same sample after phobleaching of part of the adsorbed layer. e) Quantification of c) and d). The raw signal was calculated by considering the dotted blu region in panel c), the background was calculated in the bleached region (dotted line in d). Subtracting from the raw signal the background we obtained the signal coming only from the adsorbed proteins.
(0.32 MB PDF)

Figure S8 AFM images of surface morphology before and after BSA adsorption. a) Sample after incubation with BSA solution at 3.5 µM, a surface roughness of 25.4±0.1 nm shows that after adsorption at low concentration, surface morphology is not substantially changed. (b) Sample after incubation with BSA 27.5 µM, which causes a remarkable surface flattening, resulting in a surface roughness of 17.2±0.1 nm. Colour scale range is 0–120 nm (black to white).
(1.35 MB PDF)

Figure S9 Quantitative AFM analysis scheme. The pore finding procedure is schematically represented. The objective of the analysis is to find surface pores and to measure pores width and depth. Pore dimensions depend on the surface height where dimensions are evaluated. As an example P1 has a width of 145 nm if measured at z1 = 20 nm, but it has a width of 25 nm when measured at z3 = −20 nm (pore P7). Along a surface profile the pore finding algorithm is repeated for different values of z from zmax to zmin (the maximum and the minimum surface height respectively) with a step of 2 nm. In the sketched example we simplified the procedure considering only 4 steps: a) zmax = 40 nm, b) z1 = 20 nm, c) z2 = 0 nm, d) z3 = −20 nm. a) For zmax no pores are found. b) P1 and P2 pores are found at z1. c) For z2 four pores are found. d) For z3 pores P7 and P8 are found. Only pores P1, P2, P3 and P6 will be used for statistical analysis because P4, P5, P7 and P8 are part of bigger pores P1, P2, P1 and P6 respectively.
(0.23 MB PDF)

Figure S10 L_max_ calculation. L_max_ is the width of the largest pore that is filled by proteins. In order to measure L_max_ we calculated the difference, ΔN, between the number of pores before and after adsorption of fibrinogen at 27.5 µM as a function of the maximum pore width used for the morphology analysis. L_max_ was chosen as the threshold beyond which ΔN becomes constant, approximately 150 nm.
(0.02 MB PDF)

Figure S11 AFM quantitative images analysis for BSA adsorption. (a) Widths spectrum of pores after sample incubation with PBS, BSA at 3.5 µM and 27.5 µM. (b) Depths spectrum of pores after sample incubation with PBS, BSA at 3.5 µM and 27.5 µM. Depth distribution after adsorption at 27.5 µM is very different from the other two in the whole depth range. In the region between 50 nm and 100 nm population is completely depleted, on the other hand spectrum shows a higher population in the region 0 nm–40 nm. (c) Aspect ratios spectrum of pores after sample incubation with BSA at 3.5 µM and 27.5 µM. For aspect ratio higher than 0.4 the 75% of pores are filled, showing that nucleation preferentially occurs in pores with high aspect ratio.
(0.13 MB PDF)

Figure S12 Pores volume and protein cluster dimension. (a) Total volume and total volume normalized for the sample specific area for pores in a µm^2^ with aspect ratio higher than 0.5 as a function of surface roughness. The dotted line indicates the trend follow by the normalized volume if it were proportional to the specific area. The pores volume increases beyond the increase of the specific area. (b) Number of protein clusterization sites per µm^2^ as a function of surface roughness. (c) Protein clusters mean dimension as a function of surface roughness. By increasing surface roughness, a significant increase of adsorbed proteins was observed because of the increase of the number of protein nuclei and due to the increase of their dimension. Error bars correspond to standard deviation of 3 experiment replicates.
(0.03 MB PDF)
