# Supplementary material for: The Effect of Surface Nanometre-Scale Morphology on Protein Adsorption
Source: PLoS One. 2010 Jul 29;5(7):e11862. doi: 10.1371/journal.pone.0011862 (PMC2912332; doi:10.1371/journal.pone.0011862)
Supplement: Supporting Discussion S1 — The AFM measurement of the available area for protein adsorption, PSIM fluorescence linearity, protein immobilization, fluorescence photobleaching quantification and the properties of the proteins used in this study are discussed in further details. (0.28 MB PDF) [file pone.0011862.s001.pdf]

# SUPPORTING DISCUSSION

# The effect of surface nanometre-scale morphology on protein adsorption

*Pasquale Emanuele Scopelliti[1,2*], Marco Indrieri[1], Antonio Borgonovo[1,2], Luca Giorgetti [1,3],*

*Gero Bongiorno[1,2], Roberta Carbone[4], Alessandro Podestà[1], Paolo Milani[1,2]*.*

[1] Interdisciplinary Centre for Nanostructured Materials and Interfaces (CIMaINa) and Physics Department, Università degli studi di Milano, Milan, Italy

[2] Fondazione Filarete, Milan, Italy

[3] Department of Experimental Oncology, European Institute of Oncology Campus IFOM-IEO, Milan, Italy.

[4]Tethis srl, Milan, Italy

*Corresponding author: paolo.milani@mi.infn.it

**INDEX**

AFM measurement of the available area for protein adsorption . . . . . . . . . . . . . . . . . . . . . . . . . . . . 2

PSIM: fluorescence signal linearity . . . . . . . . . . . . . . . . . . . . . . . . . . . . . . . . . . . . . . . . . . . . . . . 4

PSIM: protein immobilization . . . . . . . . . . . . . . . . . . . . . . . . . . . . . . . . . . . . . . . . . . . . . . . . . . . . . 5

Protein properties . . . . . . . . . . . . . . . . . . . . . . . . . . . . . . . . . . . . . . . . . . . . . . . . . . . . . . . . . . . . . . 5

Fluorescence Photobleaching Quantification . . . . . . . . . . . . . . . . . . . . . . . . . . . . . . . . . . . . . . . . . 6

**AFM MEASUREMENT OF THE AVAILABLE AREA FOR PROTEIN ADSORPTION**

We implemented numerical simulations in order to study the effect of AFM tip-surface convolution on the measurement of ns-TiOx samples specific area (Fig. S2). With these simulations we demonstrate that the convolution effects do not influence the measurement of relative increase of specific area (i.e the ratio between the specific area of two samples) (Fig. S3.d). Moreover, since the AFM tips have dimensions (5 nm – 7 nm) very similar to the typical dimensions of proteins used in our experiments (3 nm – 10 nm), the maximum error in the measurement of the absolute value of surface area available for protein adsorption is estimated to be 8.5% (Fig. S3.b), while on average this error is below 3.5%.

**Surface simulation**

It is well known that a wide class of processes, such as plots of random walks, interfaces in far-from-equilibrium systems and interfaces resulting from growth processes, lead to objects with self-affine properties [1, 2]. In particular the surface morphology of films deposited by SCBD has been shown to evolve with the deposition time (film thickness) according to simple scaling laws. These surfaces belong to the class of self-affine fractal surfaces [3, 4, 5]. The in-plane correlations of self-affine surfaces (or profiles) are described by two exponents: the Hurst exponent *H* and the correlation length ξ, which is the characteristic length over which two randomly chosen points on the surface (or on the profile) have uncorrelated heights. The average quadratic difference between heights of two points separated by a distance Δx (also called the height-height correlation function) scales as $\Delta x^{2H}$ for $\Delta x < \xi$, then it saturates [2]. In the case of ns-TiOx samples 1-5 (Fig. 1 and Table 1, roughness 15 – 30 nm), from the analysis of AFM topographies we have obtained a value for the roughness exponent *H* of approximately 0.8, and we have measured correlation lengths *ξ* ranging from ~30 nm to ~50 nm. In order to simulate ns-TiOx profiles, we generated statistically self-affine profiles using the random midpoint displacement algorithm [6, 7]. In particular we created sampled self-affine profiles with *H* of 0.8 and roughness and ξ similar to the experimental data for samples 1, 3 and 5 (simulated surfaces SIM1, SIM3 and SIM5 respectively, Fig. S2.g). For each set of H, ξ and roughness, we generated 100 profiles with $2^{13}$ points and with a spatial length of 2 µm.

**Specific area definition**

For a topographic map consisting in an array of N height values $\{h_{ij}\}$, the specific area $S_{A3D}$ is the ratio of the three-dimensional area calculated on the image to the projected area, i.e. the AFM scanning area. It is calculated as:

$$S_{A3D} = \frac{1}{N}\sum_{ij}\sqrt{1+\left|\nabla h_{ij}\right|^2},$$

where $\left|\nabla h_{ij}\right|$ is the squared modulus of the surface gradient. For a profile, the specific area $S_{A2D}$ is the ratio of the effective length to the projected length on x-axis direction. It is calculated as:

$$S_{A2D} = \frac{1}{l}\sum_i \sqrt{dx_i^2 + dh_i^2}\,.$$

The roughness of ns-TiOx films is calculated from AFM maps as the root-mean-squared deviation of surface heights from the average value.

**Probes convolution**

To study the effect of tip-surface convolution on the measurement of the specific area available for protein adsorption we implemented an algorithm for studying the convolution between simulated profiles and AFM-like and protein-like probes. We generated AFM-like probes as cones with half-angle θ=10° truncated and capped by a sphere of radius R of 5 nm and 7 nm (TIP5 and TIP7), accordingly. We have obtained these probes using the following expression [8]:

$$P(x) = \begin{cases} z_0 - \sqrt{R^2 - (x - x_0)^2} & for \quad (x - x_0) \le R\cos(\vartheta) \\ z_0 - \dfrac{R}{\sin(\vartheta)} + \cot an(\vartheta)(x - x_0) & for \quad (x - x_0) > R\cos(\vartheta) \\ z_0 - \dfrac{R}{\sin(\vartheta)} - \cot an(\vartheta)(x - x_0) & for \quad (x - x_0) < -R\cos(\vartheta) \end{cases},$$

where $(x_0,z_0)$ are the coordinates of the centre of the sphere. For generating probes similar to proteins (protein-like probes), we took into account the dimensions of the proteins used in our experiment and we generated probes of half-angle θ=0.1° with a curvature radius of 3 nm, 5 nm, 7 nm and 10 nm (PROT3, PROT5, PROT7 and PROT10. These values are in the range of the Stoke radii of the proteins we used in the experiments: 3.8 nm for BSA and 10.7 nm for fibrinogen). It is well known that the geometrical distortion caused by the tip to the data is not related to Fourier convolution, which is linear and invertible, but is related to the dilation algorithm, also known as Minkowsky addition [9, 10]. In particular, using custom routines and the dilation algorithm of the Image Processing Toolbox of Matlab (MathWorks), we have obtained the convoluted profiles by means of a translation of the structuring element (the tip) over the original self-affine profiles. In order to obtain a spatial resolution similar to the experimental data (0.98 nm/pixel), after performing the convolution between the simulated profiles and the tips, we down-sampled to 2048 points each profiles.

In Fig. S2 the experimental profile of sample 5 (SMP5, Fig. S2.a,d) is compared with a representative simulated profile SIM5 (Fig. S2.c,f) and to the simulated profile SIM5 after convolution with AFM tip of 5 nm radius (TIP5, Fig. S2.b,e) on different length scales (on the scale of 2 μm in the left panels and of 500 nm on the right panels). SIM5 profile after convolution and a representative experimental AFM profile are very similar. This observation is confirmed by the comparison of the $S_{A2D}$ values, showing that the specific areas of the convoluted SIM1 (SIM1_TIP5), SIM3 (SIM3_TIP5) and SIM5 (SIM5_TIP5) profiles are close to the values measured for ns-TiOx samples 1 (SMP1), 3 (SMP3) and 5 (SMP5), respectively (Fig. S2.g).

**Results**

We performed the convolution between the simulated surfaces and different probes in order to compare the effect of probe dimension on the measurement of the surface area (Figure S3.a). We used AFM-like probes of 5 nm and 7 nm curvature radius, and protein-like probes with radius between 3 nm and 10 nm. We calculated the difference between specific area measured by the two AFM tips and the available area for protein adsorption for protein probes of different dimensions (Fig. S3.b).

The difference between the measured surface area and the area at the atomic scale level ($S_{A2D}$) is between 23% and 35%. However, being the dimension of the AFM tips and proteins on the same length scale, the difference between the specific area measured by AFM and that available for protein adsorption is at most 8.5%, and on average below 3.5%. Moreover, increasing surface roughness, the AFM specific area and the area measured with different protein probes follow very similar linear trends (Fig. S3.c). Because of this similarity, taking into account the relative increase of surface area (i.e the ratio between SIM5 and SIM1 area), the difference between the measurements of AFM-like and protein-like probes is at most of 2.2%. Remarkably, the AFM measurement of the relative increase of surface area is very similar also to the same measurement at the atomic scale level, with a maximum discrepancy of just 2.8%.

**PSIM: FLUORESCENCE SIGNAL LINEARITY**

Even if there are several techniques for studying proteins adsorption that use labeled proteins such as total internal reflection fluorescence (TIRF) [11, 12], as far as we know, the problem of the linearity between the fluorescent signal and the amount of adsorbed proteins has never been coped with systematically. Using PSIM we developed an assay that allows checking for the possible influence of the fluorescent marker and for the linearity of the fluorescent signal as a function of the amount of adsorbed proteins (Fig. S4.a). We prepared an array composed of 4 sub arrays of 6 lines (10 replicates per line). In each sub-array we maintained constant the protein concentration but we varied the part of the proteins that were labeled. In different sub-arrays we tested different protein concentrations (0.75 µM, 1.5 µM, 3 µM e 6 µM) in order to check for concentration dependent effects (Fig. S4.b). Since in a sub-array the total protein concentration is kept constant we expect to have the same amount of adsorbed proteins along sub-array lines and the fluorescent signal should scale linearly with the part of labeled proteins. Any deviation from this behavior can be attributed to a different interaction of labeled proteins compared with not-labeled ones or to a non-linearity of the fluorescent signal. The relation between the part of labeled proteins and the fluorescent signal is linear in the whole concentration range considered in the assay (Fig. S4.c). This result demonstrates that the fluorescence signal is proportional to the amount of adsorbed proteins and also that the used dye does not influence protein-surface interaction. This essay exploits PSIM high-throughput

capability of testing different proteins solutions on the same sample and it allows verifying the linearity of the fluorescence signal in a fast, easy and reliable way.

**PSIM: PROTEIN IMMOBILIZATION**

We tested protein stability on the time scale of the washing steps of PSIM experiments (6 minutes), using Fluorescence Recovery After Photobleaching (FRAP). This technique allows monitoring the rate exchange of immobile proteins and measuring the immobile/mobile part ratio [13, 14]. Fluorescently labeled BSA at 8 μM was incubated with ns-$TiO_x$ titania samples for 1 h. Part of the adsorbed layer was bleached with an intense laser pulse and the fluorescence recovery in the bleached part was measured every 10 minutes (Fig. S6) the same settings for bleaching and imaging used for FPQ experiments were used (see Methods). FRAP data show that almost 100% of the adsorbed proteins are immobilized in the time scale of the PSIM experiment, and this result is surface morphology independent (Fig. S6).

**PROTEIN PROPERTIES**

Bovine Serum Albumin (BSA) is a large globular protein with molecular weight of 66 KDa [15]. It is widely used as model protein for studying the adsorption process because it belongs to the class of Albumin proteins, which constitute more than 50% of proteins in plasma, and because of its availability, ready solubility and solution stability. It is generally used as carrier for conjugation in antibody production, for blocking non-specific binding sites in immunochemical and immunoblot applications such as ELISA or Western Blot. BSA physical ancd chemical properties have been widely characterized [15-18]; BSA is made by a single polypeptide chain, consisting of about 583 amino acid residues and no carbohydrates. At pH 5-7 it contains 17 intrachain disulfide bridges and 1 sulfhydryl group [15, 17]. Its PI is 4.7 in water at 25°C [19, 20]. Its overall dimensions are 4 x 4 x 14 nm (ellipsoid) and it has a Stokes radius of 3.83 nm [21].

Fibrinogen, or Factor I, is a soluble plasma glycoprotein synthesized by the liver with a molecular weight of approximately 340 KDa. The study of fibrinogen adsorption is particularly relevant because it is the most important protein of the blood coagulation system, being responsible for blood clotting. Fibrinogen is a large protein and it consists of two identical subunits that contain three polypeptide chains: alpha, beta and gamma. The α-chain has an approximate molecular weight of 63.5 KDa, the β-chain 56 KDa, and the γ-chain 47 KDa [22-24]. Fibrinogen is converted by thrombin into fibrin, which, by means of a polymerization, forms a fibrin clot. Fibrinogen clotting underlies pathogenesis of MI, thromboembolism and thromboses of arteries and veins, since fibrin is the main substrate for thrombus formation. Fibrinogen activation is also involved in pathogenesis of inflammation, tumor growth and many other diseases [25-26]. Fibrinogen proteins have a rod-like shape with dimensions of 6 x 9 x 45 nm, and a Stokes radius of about 10.7nm.

Streptavidin is a tetrameric protein (molecular weight = 52 KDa), with overall dimensions of 4.5 x 5 x 5.5 nm [27]. It is isolated from the actinobacterium Streptomyces avidinii. This protein is remarkable for its extraordinarily strong affinity for biotin (binds up to four molecules of biotin with an association constant of about $10^{-15}$ M) [28, 29]. This property makes the investigation of streptavidin adsorption of great interest for many applications. In fact, streptavidin is extensively used as probe in immunochemical systems, conjugated to antibodies, enzymes or fluorochromes for detection and purification of various biomolecules.

**FLUORESCENCE PHOTOBLEACHING QUANTIFICATION**

We used confocal microscope for reproducing PSIM results and we developed a quantitative method for measuring the amount of adsorbed proteins. Confocal microscope allows imaging the fluorescence of the protein layer in presence of protein solution, giving the possibility to perform quantitative measurements (Figure 4). Since the confocal microscope resolution (500 – 1000 nm with settings used for our experiments) is well above the dimension of the protein layer (10 - 100 nm), the measured signal (raw signal) is the sum of the adsorbed protein layer signal and the signal coming from proteins present in solution in the resolution volume (background signal). In order to isolate the fluorescence signal of adsorbed proteins the correct measurement of the background profile is needed. Background profile has a complex shape in correspondence of the interface between the nanostructured material support (glass coverslip) and the protein solution. It is indeed affected by point spread function (PSF) convolution effects and by optical aberrations caused by the refractive index mismatch between PBS and glass. Spherical aberration produces fluorescence intensity loss along the z axis and the effect increases rapidly with the refractive index difference and with the distance of the object from the support (nominal focal position, NFP) [30]. In a mismatched system, the only part of the specimen that can be observed without the severe effects described above is the one immediately next to the coverslip. As a consequence, background quantification is not straightforward. We have verified the presence of this two effects using BSA Alexa 647 conjugated incubated with an antifouling surface (glass coverslip passivated with BSA) (Fig. S7.a). In this experiment the expected ideal signal is a step function (dotted line in Fig. S7.b): the profile signal in correspondence of the interface should rapidly change from the noise level to the background level. However, as mentioned above, the real signal is affected by PSF convolution, which converts the step function in “S” shape function, and interface optical aberration, which causes the drop of the background signal along the z axis (Fig. S7.b). These effects make the background profile quite complex in correspondence of the interface where it is located the adsorbed proteins layer. It is in principle possible to measure the background profile with a calibration measurement using a passivated surface as shown in Fig. S7.a. However this would require a preliminary measurement and would reduce the freedom of optimizing imaging settings.

We developed a new simple approach based on fluorescence photobleaching, which allows measuring, on the same image, the raw signal and the background. Using high laser intensity we bleached a small region of the adsorbed protein layer (approximately 225 $\mu m^2$). This allows isolating and measuring the background profile in a part of the image (Fig. S7.c and S7.d). Subtracting the background profile to the raw signal it is possible to obtain the fluorescent signal coming from the adsorbed proteins (L), which, has expected, has a PSF like shape (Fig. S7.e).

To quantify the amount of adsorbed proteins (ρ) we consider the resolution volume of the microscope (i.e. the minimum resolvable volume in a specific configuration, V), which in our case is 1$\mu m^3$, the protein concentration in solution (C), which should be measured using a spectrophotometer, and the fluorescence intensity of the background (BG). The density of adsorbed proteins on the surface, in proteins/$\mu m^2$, can be calculated as:

$$\rho = \frac{L}{BG}\frac{C\cdot V}{V^{2/3}}.$$

We used this approach because it has not limitations related to surface roughness as other quantitative techniques have, such as quartz crystal microbalance (QCM) or ellipsometry, and because it allows an easy quantification based on a single fluorescence measurement.

## REFERENCES

[1] Moreira, J.G., Leal da Silva, J. K. & Kamphorst, S.O. On the fractal dimension of self-affine profiles. *J. Phys. A: Math. Gen.* **27,** 8079-8089 (1994).

[2] Barbasi, A. L. & Stanley, H. E. *Fractal concepts in surface growth* (Cambridge Univ. Press, New York, 1995).

[3] Milani, P. *et al*, C. Cluster assembling of nanostructured carbon films. *Diamond Relat. Mater.* **10,** 240-247 (2001).

[4] Podestà, A. *et al* Cluster-Assembled Nanostructured Titanium Oxide Films with Tailored Wettability. *J. Phys. Chem. C* **113,** 18264-18269 (2009).

[5] Buzio, R. *et al* Self-affine properties of cluster-assembled carbon thin films. *Surface Science Letters* **444,** L1-L6 (2000).

[6] Schmittbuhl, J., Vilotte, J.P. & Roux, S. Reliability of self-affine measurements. *Phys. Rev. E* **51,** 131-147 (1995).

[7] Castelnovo, C., Podestà, A., Piseri, P. & Milani P. Fractal analysis of sampled profiles: Systematic study. *Phys. Rev. E* **65,** 021601-1 - 021601-11 (2002).

[8] Odin, C., Aime', J.P., El Kaakour, Z. T., & Bouhacina, T. Tip's finite size effects on atomic force microscopy in the contact mode: simple geometrical considerations for rapid estimation of apex radius and tip angle based on the study of polystyrene latex balls. *Surface Science* **317,** 321- 340 (1994).

[9] Villarrubia, J. S. Algorithms for Scanned Probe Microscope Image Simulation, Surface Reconstruction, and Tip Estimation *J. Res. Natl. Inst. Stand. Technol.* **102,** 425-545 (1997).

[10] Castle, J.E., Zhdan, P.A. & Singjai, P. Enhanced morphological reconstruction of SPM images. *J. Phys. D: Apll. Phys*. **31,** 3437-3445 (1998).

[11] Toscano, A. & Santore, M. M. Fibrinogen Adsorption on Three Silica-Based Surfaces: Conformation and Kinetics. *Langmuir* **22,** 2588–2597 (2006).

[12] Mollmann, S. H. *et al* Interfacial adsorption of insulin Conformational changes and reversibility of adsorption. *European Journal of Pharmaceutical Sciences* **27,** 194–204 (2006).

[13] Carrero, G. Using frap and mathematical modeling to determine the in vivo kinetics of nuclear proteins. *Methods* **29,**14–28 (2003).

[14] Vink, M. *et al* In vitro frap identifies the minimal requirements for mad2 kinetochore dynamics. *Current Biology* **16,** 755–766 (2006).

[15] Hirayama, K., Akashi, S., Furuya, M. & Fukuhara, K. Rapid confirmation and revision of the primary structure of bovine serum albumin by esims and frit-fab lc/ms. *Biochemical and biophysical research communications*. **173,** 639–646 (1990).

[16] Fudenberg, H. *The plasma proteins: Structure, function, and genetic control* (Frank w. Putnam. academic press, New York, 1975).

[17] Reed, R. G., Putnam, F. W. & Peters. T. Sequence of residues 400–403 of bovine serum albumin. *Biochemical Journal* **191,** 867–875 (1980).

[18] Reed, R., G., Feldhoff, R., C. & Peters, T. Fragments of bovine serum albumin produced by limited proteolysis: complementary behavior of two large fragments. *Biochemistry* **15,** 5394–5398 (1976).

[19] Dawson, R., M., C., Elliott, D., C., Elliott, W.,H. & Jones, K. M. *Data for Biochemical Research* (Oxford Univ. Press, USA, 1986).

[20] Malamud, D. & Drysdale, J. Isoelectric points of proteins: A table. *Analytical Biochemistry* **86,** 620–647 (1978).

[21] Righetti, P. G. & Caravaggio, T. Isoelectric points and molecular weights of proteins. *Journal of chromatography* **127,** 1–28 (1976).

[22] Doolittle, R. F. The structure and evolution of vertebrate fibrinogen. *New York Academy Sciences Annals* **408,** 13–27 (1983).

[23] Henschen, A., Lottspeich, F., Kehl, M. & Southan, C. Covalent structure of fibrinogen. *Annals of the New York Academy of Sciences* **408,** 28–43 (1983).

[24] Doolittle, R., F. Fibrinogen and fibrin. *Annual review of biochemistry* **53,** 195–229 (1984).

[25] Bark, N. On the occurrence of thrombin-like enzymes in mosquitoes. *Thrombosis Research* **81,** 623–634 (1996).

[26] Blombäck, M., Blombäck, B., Mammen, E., F. & Prasad, A., S. Fibrinogen detroit–a molecular defect in the n-terminal disulphide knot of human fibrinogen? *Nature* **218,** 134–137 (1968).

[27] Chaiet, L., & Wolf, F., The properties of streptavidin, a biotin-binding protein produced by. *Archives of Biochemistry and Biophysics* **106,** 1–5 (1964).

[28] Darst, S., Two-dimensional crystals of streptavidin on biotinylated lipid layers and their interactions with biotinylated macromolecules. *Biophysical Journal* **59,** 387–396 (1991).

[29] Jencks, W., P. On the attribution and additivity of binding energies. *Proceedings of the National Academy of Sciences of the United States of America* **78,** 4046–4050 (1981).

[30] Pawley, J. *Handbook of Biological Confocal Microscopy* (Springer, 2006).
