# Supplementary material for: The Effect of Surface Nanometre-Scale Morphology on Protein Adsorption
Source: PLoS One. 2010 Jul 29;5(7):e11862. doi: 10.1371/journal.pone.0011862 (PMC2912332; doi:10.1371/journal.pone.0011862)
Supplement: Supporting Methods S1 — Supporting Methods. (0.13 MB PDF) [file pone.0011862.s002.pdf]

SUPPORTING METHODS

The effect of surface nanometre-scale morphology on protein adsorption

Pasquale Emanuele Scopelliti[1,2*] *, Marco Indrieri*[1]*, Antonio Borgonovo*[1,2]*, Luca Giorgetti* [1,3]*,*

Gero Bongiorno[1,2]*, Roberta Carbone*[4]*, Alessandro Podestà*[1]*, Paolo Milani*[1,2]**.*

[1] Interdisciplinary Centre for Nanostructured Materials and Interfaces (CIMaINa) and Physics Department, Università degli studi di Milano, Milan, Italy

[2] Fondazione Filarete, Milan, Italy

[3] Department of Experimental Oncology, European Institute of Oncology Campus IFOM-IEO, Milan, Italy.

[4]Tethis srl, Milan, Italy

*Corresponding author: paolo.milani@mi.infn.it

Sample morphology characterization with AFM

The investigation of morphology of the substrates was carried out in air using a Multimode AFM equipped with a Nanoscope IV controller (Veeco Instruments). The AFM was operated in Tapping Mode use single crystal silicon tips with nominal radius of curvature 5-10 nm and cantilever resonance frequency in the range of 200-300 kHz. Scan areas were 2µm x 1µm with scan rates of 1.5-2 Hz. Sampling resolution was 2048x512. Typically at least three images were acquired on each sample. AFM images were processed using custom routines written in a Matlab environment. Each AFM-image was flattened, subtracting a global plane and line-by-line first - or occasionally second - polynomials in order to get rid of the tilt of the sample and of the scanner bow. The RMS roughness σ is calculated from $\sigma = \sqrt{\frac{1}{N}\sum_{i,j}(h_{ij} - \bar{h})^2}$, where h_{ij} represents the height values (*i,j* are the row, column indices) and *N* the number of height values in the AFM topographic map, $\bar{h}$ is the average height ($\bar{h} = \frac{1}{N}\sum_{i,j} h_{i,j}$). The effective or specific area A_{eff} is the ratio of the three-dimensional area calculated on the image to the projected area, i.e. to the AFM scanning area. It is calculated as $A_{eff} = \frac{1}{N}\sum_{ij}\sqrt{1 + \left|\nabla h_{ij}\right|^2}$, where ∇h_{ij} is the surface gradient vector.

RSA theoretical monolayer density calculation

In order to calculate protein monolayers, we exploited the Random Sequential Adsorption theory (RSA). We defined the maximum packing for RSA (i.e. jamming limit) as a full monolayer, which corresponds to a coverage of about 56% of the available surface. For each protein we calculated the two limit values for

side-on and end-on adsorption cases. In order to have a unique average value for protein monolayer, we calculated the weighted average of the two limit values, considering as weights the inverse of the superficial area of each adsorption side, reflecting the likelihood of each adsorption case. Finally these values were multiplied by the specific area (SA) of each sample, for taking into account the SA contribution.

AFM protein adsorption analysis

The morphology of each sample was acquired before adsorption. The sample was then incubated with 400 μL of proteins solution for 1h without removing it from the AFM stage. Samples were washed 3 times for 1 minute in PBS and 3 times in bidistilled H_2O for 1 minute. Samples were dried using a gentle nitrogen flux. Surface morphology was acquired before and after proteins adsorption in air using a Bioscope II AFM equipped with a Nanoscope V controller (Veeco Instruments). The AFM was operated in Tapping Mode use single crystal silicon tips with nominal radius of curvature 5-10 nm and cantilever resonance frequency in the range of 200-300 kHz. Scan areas were 2μm x 1μm with scan rates of 1.5-2 Hz.

AFM quantitative image analysis

AFM quantitative analysis was performed using custom routines written in a Matlab environment. For finding pores, the surface profile was scanned several times, changing the xy plane height at which the pore width was calculated (starting from z_{max} to z_{min} with a step of 2 nm, Fig. S9). When a pore was found, its width, height and aspect ratio were included in the spectra if it had a width lower than a threshold value, L_{max}, and higher than 10 nm, and if it was not part of another pore already found in a previous scan with higher z value (Fig. S10). L_{max} is the width of the largest pore that is filled by proteins. It was measured calculating the difference between the number of pores before and after adsorption (ΔN) as a function of the maximum pore width (Fig. S10). L_{max} is chosen as the threshold width beyond which ΔN becomes constant.
