# Supplementary figures and images for: The Effect of Surface Nanometre-Scale Morphology on Protein Adsorption
Source: PLoS One. 2010 Jul 29;5(7):e11862. doi: 10.1371/journal.pone.0011862 (PMC2912332; doi:10.1371/journal.pone.0011862)

t1

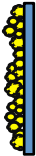

t2

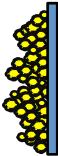

t3

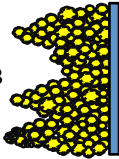

Supplement: Figure S1 — Ns-TiOx film growth. Schematic view of the film growth process as a function of the deposition time (film thickness). Changing film thickness is possible to regulate surface morphology without changing surface chemistry. This method allows varying surface roughness from 15 nm to 30 nm (Fig. 1 and table 1). (0.53 MB PDF) [file pone.0011862.s003.pdf]

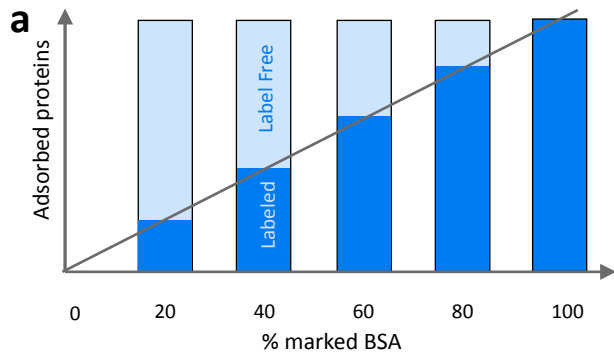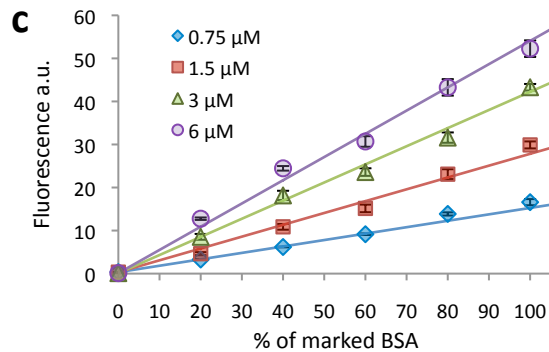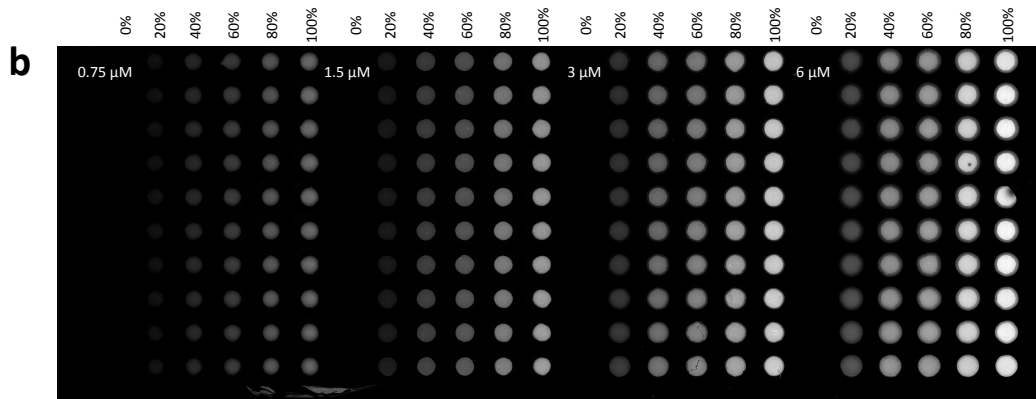

Supplement: Figure S4 — Fluorescence signal linearity. a) The sketch of the assay used to test the linearity of the fluorescent signal as a function of the amount of adsorbed proteins. The objective of the assay is to measure the fluorescent signal as a function of the part of labeled BSA in solution. b) Protein-surface interaction array composed of 4 sub-arrays; in each sub-array the protein concentration was kept constant (0.75 µM, 1.5 µM, 3 µM and 6 µM), while the part of labeled BSA in solution was varied from 0% to 100%. In each line the same BSA concentration is spotted in 10 replicates. c) Results of the PSIM validation experiment. Data follows a good linear trend for all the used concentrations, showing that the fluorescence signal is proportional to the amount of adsorbed proteins. (0.16 MB PDF) [file pone.0011862.s006.pdf]

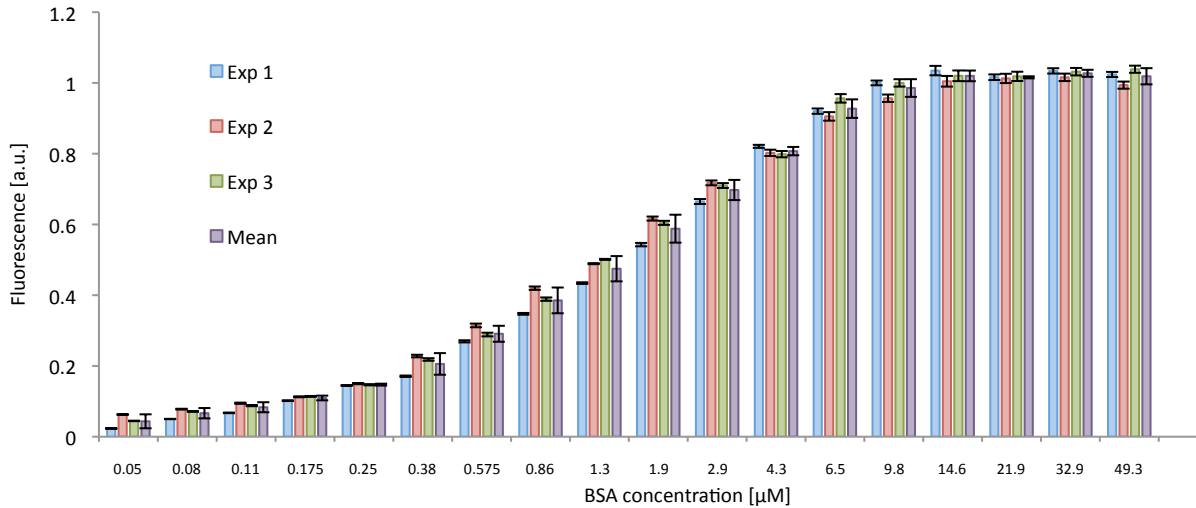

Supplement: Figure S5 — PSIM reproducibility. In order to test PSIM reproducibility we performed three independent experiments spotting 18 different fluorescently labelled BSA dilutions, in 10 replicates, on three different ns-TiOx samples (same thickness, 50 nm, resulting in a surface roughness of 15.0±0.1 and in a specific area of 1.56±0.1). The result of each experiment is compared with the mean of the three experiments (last bar of each concentration point). Error bar is the standard deviation of the 10 replicates for each experiment point. For the mean the error bar is the standard deviation of three experiments. (0.03 MB PDF) [file pone.0011862.s007.pdf]

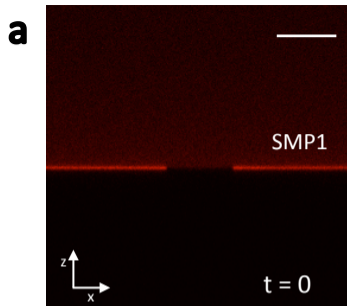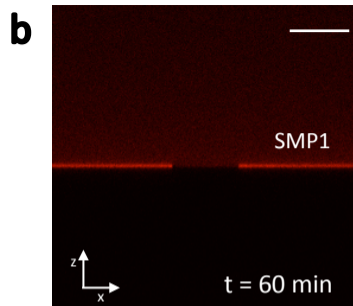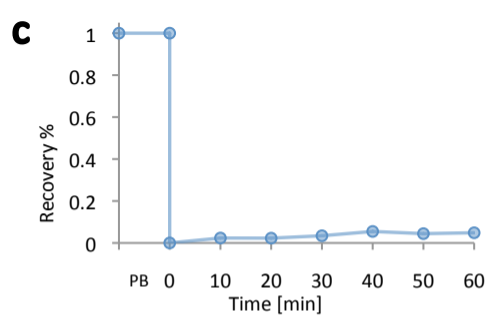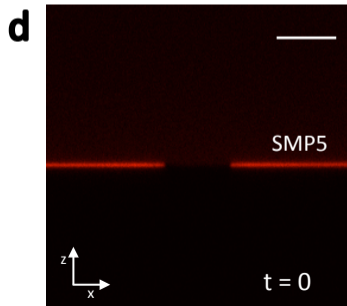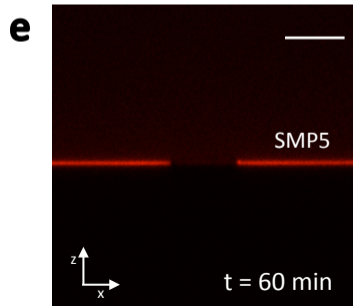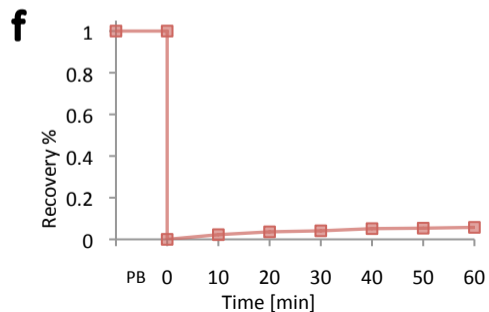

Supplement: Figure S6 — Fluorescence Recovery After Photobleaching for studying protein stability. Confocal microscope images of the adsorbed layer for sample 1 (a) just after the photobleaching of part of the adsorbed layer and and (b) 60 minutes after the photobleaching. (c) Fluorecence recovery after photobleaching as a function of time for sample 1. Confocal microscope images of the adsorbed layer for sample 5 (d) just after the photobleaching of part of the adsorbed layer and and (e) 60 minutes after the photobleaching. (f) Fluorecence recovery after photobleaching as a function of time for sample 5. (0.18 MB PDF) [file pone.0011862.s008.pdf]

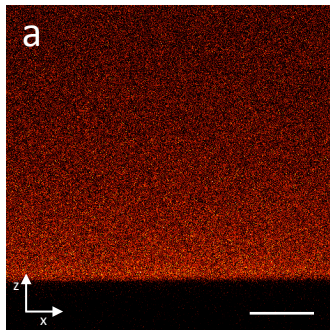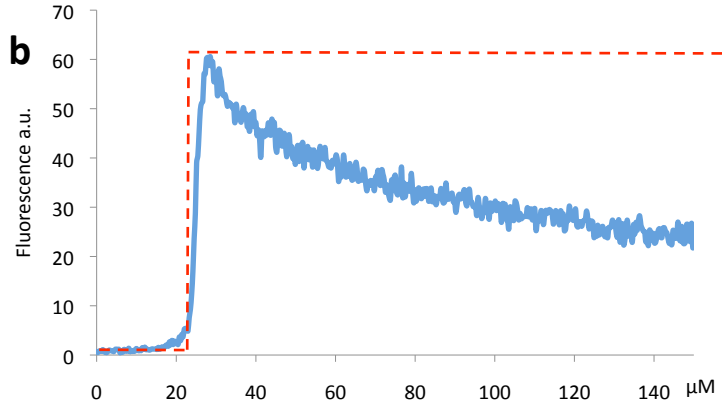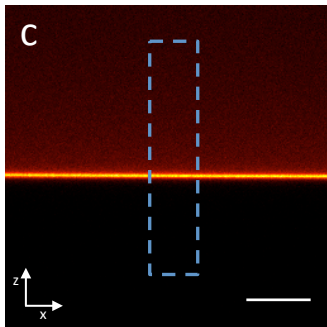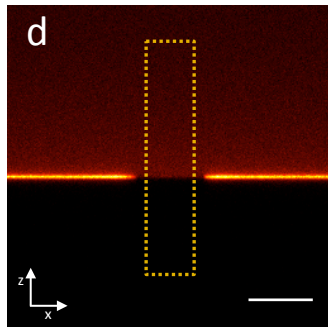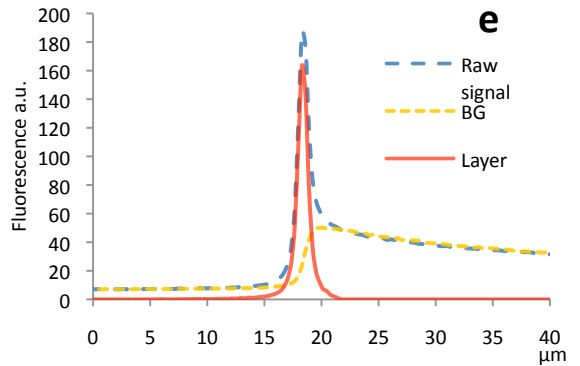

Supplement: Figure S7 — Fluorescence Photobleaching Quantification. a) A glass coverslip was passivated with BSA (without fluorescent label) in order to avoid the following adsorption of labeled proteins. Coverslip was then incubated with a solution of Fluorescent BSA 1 µM concentration. b) The the signal profile. The surface is passivated and the signal corresponds only to the BG signal, which has a complex shape because of PSF convolution and optical abberrations. The dashed line represents a step function, the expected BG shape without PSF convolution and optical aberrations. c) Image of the adsorbed layer on ns-TiOx sample incubated with fluorescent BSA 5 µM concentration. d) Image of the same sample after phobleaching of part of the adsorbed layer. e) Quantification of c) and d). The raw signal was calculated by considering the dotted blu region in panel c), the background was calculated in the bleached region (dotted line in d). Subtracting from the raw signal the background we obtained the signal coming only from the adsorbed proteins. (0.32 MB PDF) [file pone.0011862.s009.pdf]

**a**

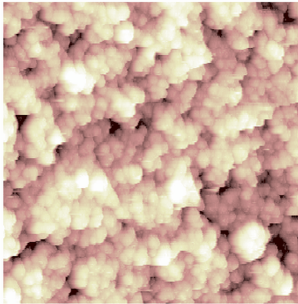

**b**

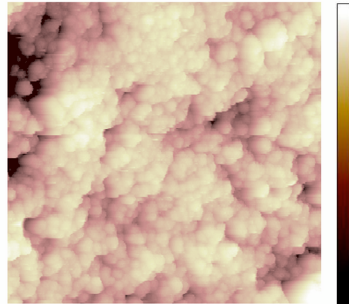

Supplement: Figure S8 — AFM images of surface morphology before and after BSA adsorption. a) Sample after incubation with BSA solution at 3.5 µM, a surface roughness of 25.4±0.1 nm shows that after adsorption at low concentration, surface morphology is not substantially changed. (b) Sample after incubation with BSA 27.5 µM, which causes a remarkable surface flattening, resulting in a surface roughness of 17.2±0.1 nm. Colour scale range is 0–120 nm (black to white). (1.35 MB PDF) [file pone.0011862.s010.pdf]

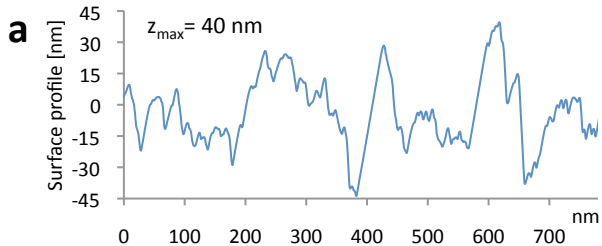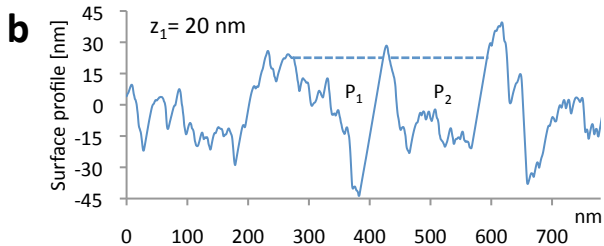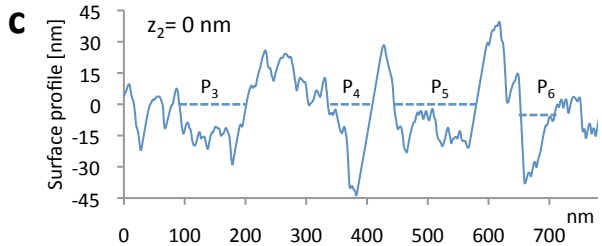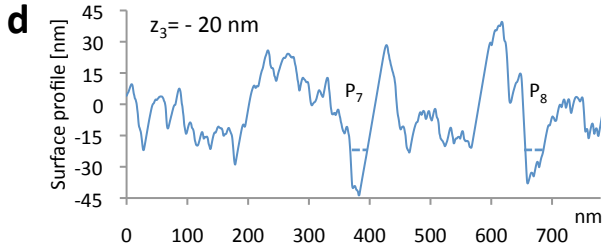

Supplement: Figure S9 — Quantitative AFM analysis scheme. The pore finding procedure is schematically represented. The objective of the analysis is to find surface pores and to measure pores width and depth. Pore dimensions depend on the surface height where dimensions are evaluated. As an example P1 has a width of 145 nm if measured at z1 = 20 nm, but it has a width of 25 nm when measured at z3 = −20 nm (pore P7). Along a surface profile the pore finding algorithm is repeated for different values of z from zmax to zmin (the maximum and the minimum surface height respectively) with a step of 2 nm. In the sketched example we simplified the procedure considering only 4 steps: a) zmax = 40 nm, b) z1 = 20 nm, c) z2 = 0 nm, d) z3 = −20 nm. a) For zmax no pores are found. b) P1 and P2 pores are found at z1. c) For z2 four pores are found. d) For z3 pores P7 and P8 are found. Only pores P1, P2, P3 and P6 will be used for statistical analysis because P4, P5, P7 and P8 are part of bigger pores P1, P2, P1 and P6 respectively. (0.23 MB PDF) [file pone.0011862.s011.pdf]

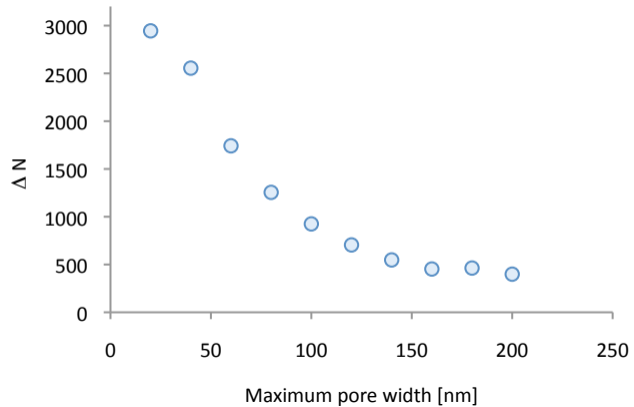

Supplement: Figure S10 — Lmax calculation. Lmax is the width of the largest pore that is filled by proteins. In order to measure Lmax we calculated the difference, ΔN, between the number of pores before and after adsorption of fibrinogen at 27.5 µM as a function of the maximum pore width used for the morphology analysis. Lmax was chosen as the threshold beyond which ΔN becomes constant, approximately 150 nm. (0.02 MB PDF) [file pone.0011862.s012.pdf]

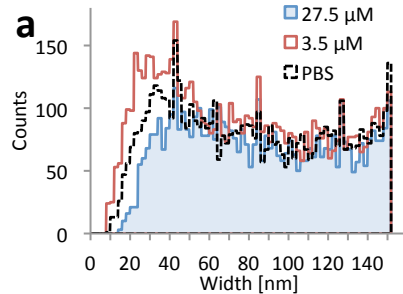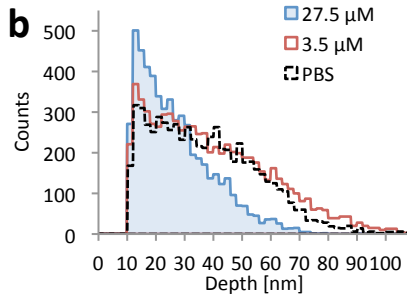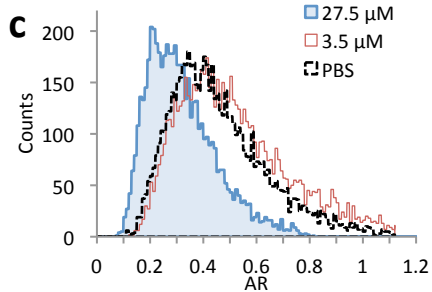

Supplement: Figure S11 — AFM quantitative images analysis for BSA adsorption. (a) Widths spectrum of pores after sample incubation with PBS, BSA at 3.5 µM and 27.5 µM. (b) Depths spectrum of pores after sample incubation with PBS, BSA at 3.5 µM and 27.5 µM. Depth distribution after adsorption at 27.5 µM is very different from the other two in the whole depth range. In the region between 50 nm and 100 nm population is completely depleted, on the other hand spectrum shows a higher population in the region 0 nm–40 nm. (c) Aspect ratios spectrum of pores after sample incubation with BSA at 3.5 µM and 27.5 µM. For aspect ratio higher than 0.4 the 75% of pores are filled, showing that nucleation preferentially occurs in pores with high aspect ratio. (0.13 MB PDF) [file pone.0011862.s013.pdf]

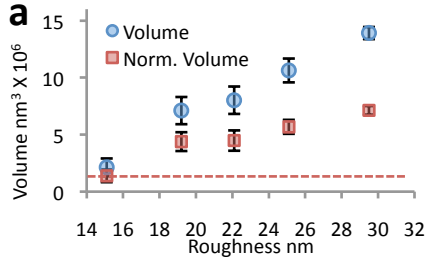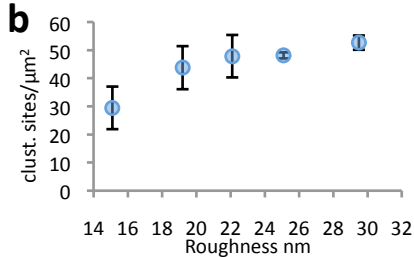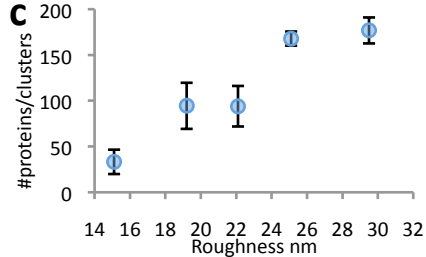

Supplement: Figure S12 — Pores volume and protein cluster dimension. (a) Total volume and total volume normalized for the sample specific area for pores in a µm2 with aspect ratio higher than 0.5 as a function of surface roughness. The dotted line indicates the trend follow by the normalized volume if it were proportional to the specific area. The pores volume increases beyond the increase of the specific area. (b) Number of protein clusterization sites per µm2 as a function of surface roughness. (c) Protein clusters mean dimension as a function of surface roughness. By increasing surface roughness, a significant increase of adsorbed proteins was observed because of the increase of the number of protein nuclei and due to the increase of their dimension. Error bars correspond to standard deviation of 3 experiment replicates. (0.03 MB PDF) [file pone.0011862.s014.pdf]
