# Supplementary material for: The Effect of Surface Nanometre-Scale Morphology on Protein Adsorption
Source: PLoS One. 2010 Jul 29;5(7):e11862. doi: 10.1371/journal.pone.0011862 (PMC2912332; doi:10.1371/journal.pone.0011862)
Supplement: Figure S2 — Simulation of the self-affine fractal ns-TiOx profile. Typical surface profile of: a) ns-TiOx sample 5 (experimental, 2 µm scale); b) SIM5_TIP5 simulated profile (SIM5) after convolution with AFM tip of radius 5 nm (2 µm scale); c) SIM5 simulated profile (2 µm scale); d) ns-TiOx sample 5 (experimental, 500 nm scale); e) SIM5_TIP5 simulated profile (SIM5) after convolution with AFM tip of radius 5 nm (500 nm scale); f) SIM5 simulated profile (500 nm scale). g) Experimental morphological parameters (left) compared with morphological parameters of simulated surfaces (center) and simulated surfaces after 5 nm tip convolution (right). The convolution of a AFM tip of radius 5 nm with simulated profiles returns a two dimensional surface area, SA2D, very similar to the experimental one, demonstrating that simulation faithfully reproduces experimental surfaces. (0.17 MB PDF) [file pone.0011862.s004.pdf]

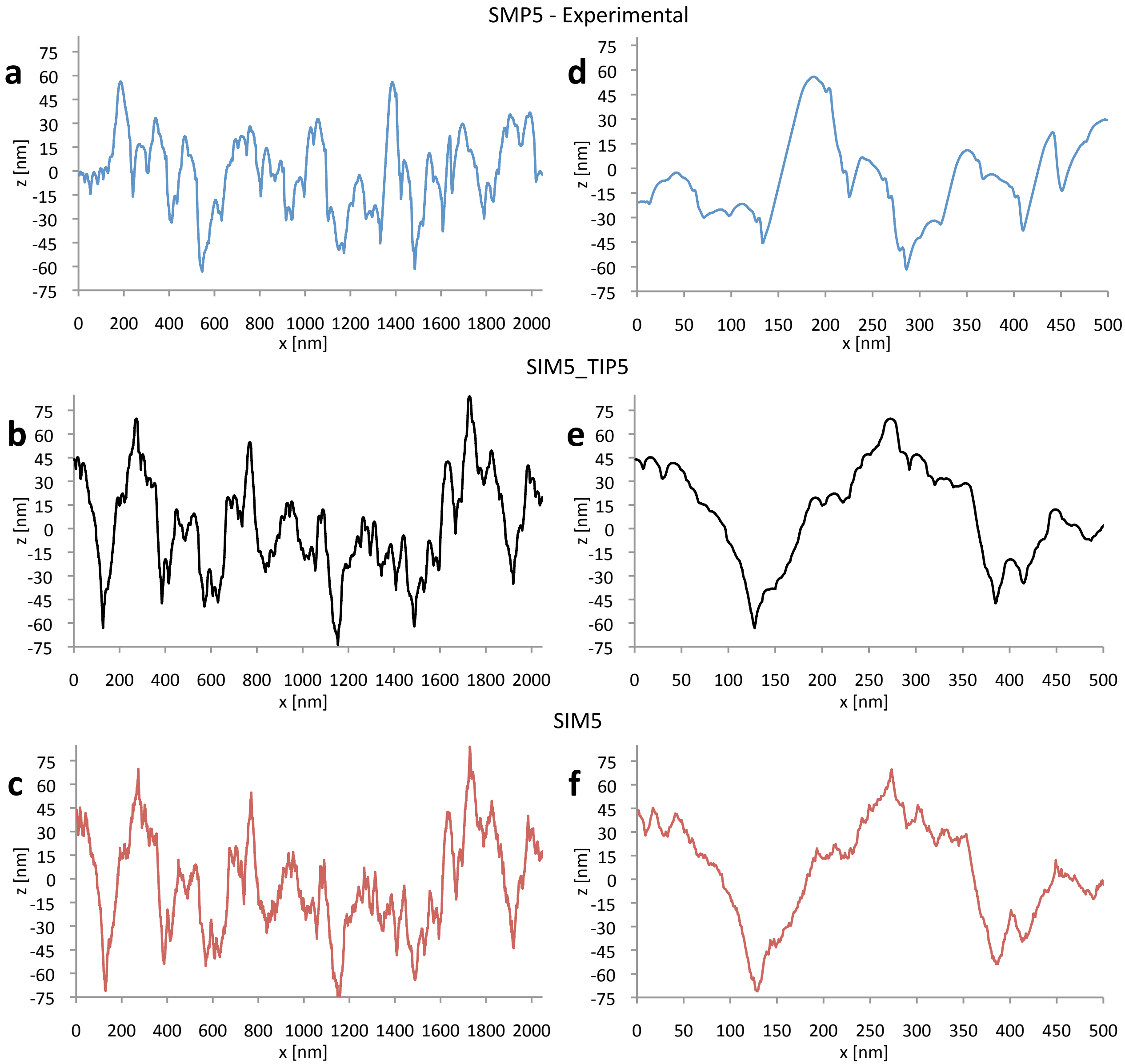

g

	Roughness	S_{A2D}
**SMP1**	15.1	1.31
**SMP3**	22.1	1.38
**SMP5**	29.5	1.56

	Roughness	S_{A2D}
**SIM1**	16	1.6
**SIM3**	22	1.66
**SIM5**	30	1.98

	Roughness	S_{A2D}
**SIM1_TIP5**	15.9	1.25
**SIM3_TIP5**	21.9	1.34
**SIM5_TIP5**	29.8	1.5
