# Supplementary material for: The Effect of Surface Nanometre-Scale Morphology on Protein Adsorption
Source: PLoS One. 2010 Jul 29;5(7):e11862. doi: 10.1371/journal.pone.0011862 (PMC2912332; doi:10.1371/journal.pone.0011862)
Supplement: Figure S3 — Surface-probes convolution results. a) The 2D surface area of simulated profiles of increasing roughness after convolution with AFM-like and protein-like probes of different radii (TIP 5 nm and 7 nm; PROT 3 nm, 5 nm, 7 nm and 10 nm). SA2D stands for the surface area of the simulated sample without any convolution. b) The relative difference between the specific area measured with AFM-like tips of radius 5 and 7 nm (left and right halves of the table) and the specific area measured with protein-like probes of different radii (mimicking the specific area available for adsorption to proteins of different dimensions). The comparison with the specific area of the not-convoluted profiles is also shown in the first raw. c) The available surface area for protein adsorption as a function of surface roughness for different probe radii. d) The relative difference between the ratios of surface areas of samples SIM5 and SIM1 measured with AFM-like and protein-like probes of different radii. (0.03 MB PDF) [file pone.0011862.s005.pdf]

a

	SIM1	SIM3	SIM5
S_{A2D}	1.597	1.656	1.983
TIP5	1.248	1.340	1.517
TIP7	1.214	1.305	1.467
PROT3	1.299	1.395	1.593
PROT5	1.251	1.342	1.518
PROT7	1.216	1.307	1.469
PROT10	1.183	1.267	1.407

b

	% TIP 5 nm			% TIP 7 nm		
	SIM1	SIM3	SIM5	SIM1	SIM3	SIM5
S_{A2D}	28.0	23.6	30.8	31.3	26.7	35.0
TIP5	0	0	0	2.6	2.5	3.3
TIP7	2.5	2.5	3.2	0	0	0
PROT3	4.1	4.1	5.1	6.9	6.7	8.5
PROT5	0.2	0.1	0.1	2.9	2.7	3.4
PROT7	2.5	2.5	3.2	0.1	0.1	0.1
PROT10	5.2	5.5	7.3	2.7	3.1	4.2

c

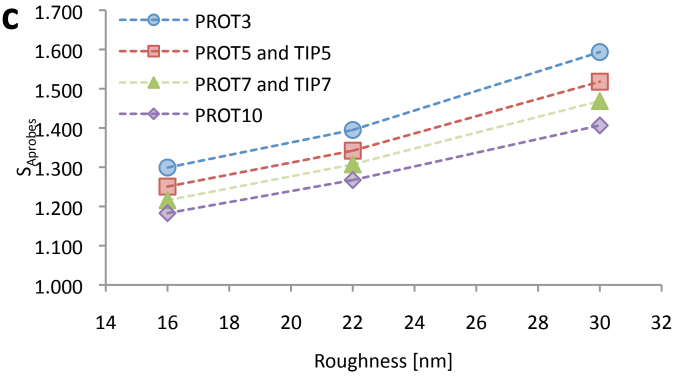

d

	TIP 5 nm	TIP 7 nm
S_{A2D}	2.2	2.8
TIP5	0	0.6
TIP7	0.6	0
PROT3	0.9	1.5
PROT5	0.2	0.5
PROT7	0.6	0.2
PROT10	2.2	1.6
